# Supplementary material for: NiCo identifies extrinsic drivers of cell state modulation by niche covariation analysis
Source: Nat Commun. 2024 Dec 5;15:10628. doi: 10.1038/s41467-024-54973-w (PMC11621405; doi:10.1038/s41467-024-54973-w)
Supplement: Supplementary file 2 — Reporting Summary [file 41467_2024_54973_MOESM2_ESM.pdf]

nature portfolio

Corresponding author(s): Dominic Grün

Last updated by author(s): Oct 10, 2024

Reporting Summary

Nature Portfolio wishes to improve the reproducibility of the work that we publish. This form provides structure for consistency and transparency in reporting. For further information on Nature Portfolio policies, see our Editorial Policies and the Editorial Policy Checklist.

Statistics

For all statistical analyses, confirm that the following items are present in the figure legend, table legend, main text, or Methods section.

n/a	Confirmed	
☐	☒	The exact sample size (*n*) for each experimental group/condition, given as a discrete number and unit of measurement
☒	☐	A statement on whether measurements were taken from distinct samples or whether the same sample was measured repeatedly
☐	☒	The statistical test(s) used AND whether they are one- or two-sided<br>*Only common tests should be described solely by name; describe more complex techniques in the Methods section.*
☒	☐	A description of all covariates tested
☒	☐	A description of any assumptions or corrections, such as tests of normality and adjustment for multiple comparisons
☐	☒	A full description of the statistical parameters including central tendency (e.g. means) or other basic estimates (e.g. regression coefficient) AND variation (e.g. standard deviation) or associated estimates of uncertainty (e.g. confidence intervals)
☐	☒	For null hypothesis testing, the test statistic (e.g. *F*, *t*, *r*) with confidence intervals, effect sizes, degrees of freedom and *P* value noted<br>*Give P values as exact values whenever suitable.*
☒	☐	For Bayesian analysis, information on the choice of priors and Markov chain Monte Carlo settings
☒	☐	For hierarchical and complex designs, identification of the appropriate level for tests and full reporting of outcomes
☒	☐	Estimates of effect sizes (e.g. Cohen's *d*, Pearson's *r*), indicating how they were calculated

Our web collection on statistics for biologists contains articles on many of the points above.

Software and code

Policy information about availability of computer code

Data collection	All data were collected from the public domain and processed using our custom-made software, NiCo. Step-by-step tutorials for NiCo (https://github.com/ankitbioinfo/nico_tutorial), detailing the processing and analysis of spatial transcriptomics and scRNA-seq data, along with its broad usage, are available here: (https://nico-sc-sp.readthedocs.io/en/latest/).
Data analysis	Data analysis was performed using our custom-designed software package, NiCo (https://pypi.org/project/nico-sc-sp/), which is also available through the link provided in the CODE Availability section of the manuscript.

For manuscripts utilizing custom algorithms or software that are central to the research but not yet described in published literature, software must be made available to editors and reviewers. We strongly encourage code deposition in a community repository (e.g. GitHub). See the Nature Portfolio guidelines for submitting code & software for further information.

Data

Policy information about availability of data

All manuscripts must include a data availability statement. This statement should provide the following information, where applicable:

- Accession codes, unique identifiers, or web links for publicly available datasets
- A description of any restrictions on data availability
- For clinical datasets or third party data, please ensure that the statement adheres to our policy

The raw data were taken from the publicly available resources with accession codes and/or web links indicated in the data availability statement.

All scRNA-seq, spatial transcriptomics, and respective cell type annotations datasets analyzed in this study are publicly available in the referenced studies. The Mouse organogenesis seqFISH data is available at https://content.cruk.cam.ac.uk/jmlab/SpatialMouseAtlas2020/, and the analogous time point of scRNAseq data can be accessed through ArrayExpress through E-MTAB-6967. The small intestine MERFISH data is available at https://datadryad.org/stash/dataset/doi:10.5061/dryad.jm63xsjb2, and the associated scRNAseq data can be found under the accession code GSE190037. The mouse liver MERFISH data is downloaded from Vizgen MERSCOPE website (https://vizgen.com/data-release-program/), while the analogous scRNAseq atlas data is available at https://www.livercellatlas.org/download.php. .The processed source data, as well as simulated, benchmark, and experimental data presented in the figures, are available at (https://zenodo.org/uploads/13895622)

Research involving human participants, their data, or biological material

Policy information about studies with human participants or human data. See also policy information about sex, gender (identity/presentation), and sexual orientation and race, ethnicity and racism.

Reporting on sex and gender	Not considered in the study design.
Reporting on race, ethnicity, or other socially relevant groupings	Not considered in the study design.
Population characteristics	Not considered in the study design.
Recruitment	Not considered in the study design.
Ethics oversight	Not considered in the study design.

Note that full information on the approval of the study protocol must also be provided in the manuscript.

Field-specific reporting

Please select the one below that is the best fit for your research. If you are not sure, read the appropriate sections before making your selection.

☒ Life sciences ☐ Behavioural & social sciences ☐ Ecological, evolutionary & environmental sciences

For a reference copy of the document with all sections, see nature.com/documents/nr-reporting-summary-flat.pdf

Life sciences study design

All studies must disclose on these points even when the disclosure is negative.

Sample size	Sample size of analyzed single cell scRNA-seq dataset is provided in the respective section of the manuscript.
Data exclusions	No data was excluded.
Replication	Experimental validation was performed in biological replicates.
Randomization	Not applicable. Experimental validation involved comparison of cells exposed to different culture condition.
Blinding	Blinding was not relevant. Experimental validation involved a statistical test between two treatment groups.

Reporting for specific materials, systems and methods

We require information from authors about some types of materials, experimental systems and methods used in many studies. Here, indicate whether each material, system or method listed is relevant to your study. If you are not sure if a list item applies to your research, read the appropriate section before selecting a response.

Materials & experimental systems

n/a	Involved in the study	
☒	☐	Antibodies
☒	☐	Eukaryotic cell lines
☒	☐	Palaeontology and archaeology
☐	☒	Animals and other organisms
☒	☐	Clinical data
☒	☐	Dual use research of concern
☒	☐	Plants

Methods

n/a	Involved in the study	
☒	☐	ChIP-seq
☒	☐	Flow cytometry
☒	☐	MRI-based neuroimaging

Animals and other research organisms

Policy information about studies involving animals; ARRIVE guidelines recommended for reporting animal research, and Sex and Gender in Research

Laboratory animals	C57B/6J males mouse with an age of 12 -16 weeks
Wild animals	Study did not involve wild animals.
Reporting on sex	Sex was not considered in the study design.
Field-collected samples	Study did not involve sample collected from the field.
Ethics oversight	All animal experiments were approved and conducted under the regulations of the local Government.

Note that full information on the approval of the study protocol must also be provided in the manuscript.

Plants

Seed stocks	Not applicable
Novel plant genotypes	Not applicable
Authentication	Not applicable
